# The Mediating Role of Perceived Social Support Between Physical Activity Habit Strength and Depressive Symptoms in People Seeking to Decrease Their Cardiovascular Risk: Cross-Sectional Study

**DOI:** 10.2196/11124

**Published:** 2018-11-14

**Authors:** Vera Storm, Dominique Alexandra Reinwand, Julian Wienert, Shu-Ling Tan, Sonia Lippke

**Affiliations:** 1 Institute of Sport and Exercise Sciences Department of Sport and Exercise Psychology University of Münster Münster Germany; 2 Faculty of Human Sciences Rehabilitative Gerontology University of Cologne Cologne Germany; 3 Scientific Institute of Techniker Krankenkasse for Benefit and Efficiency in Health Care (WINEG) Hamburg Germany; 4 Health Psychology and Behavioral Medicine Department of Psychology and Methods Jacobs University Bremen Bremen Germany; 5 Institute of Sport and Exercise Sciences Department of Social Sciences of Sport University of Münster Münster Germany; 6 Bremen International Graduate School of Social Sciences Bremen Germany

**Keywords:** physical activity, habit, social support, depressive symptoms, cardiac diseases

## Abstract

**Background:**

Regular physical activity treatment has been advocated for the prevention and rehabilitation of patients at risk of cardiovascular diseases and depressive symptoms. How physical activity is related to depressive symptoms is widely discussed.

**Objective:**

The aim of this internet-based study was to investigate the role of perceived social support in the relationship between physical activity habit strength and depressive symptoms.

**Methods:**

In total, 790 participants (mean 50.9 years, SD 12.2, range 20-84 years) who were interested in reducing their cardiovascular risk were recruited in Germany and the Netherlands. Data collection was conducted via an internet-based questionnaire addressing physical activity habit strength, depressive symptoms, and perceived social support. Cross-sectional data analysis was done with SPSS version 24 using the Macro PROCESS version 2 16.3 by Hayes with bootstrapping (10,000 samples), providing 95% CIs.

**Results:**

Physical activity habit strength was negatively related to depressive symptoms (*r*=–.13, *P*=.006), but this interrelation disappeared when controlling for perceived social support (beta=–.14, SE 0.09, *P*=.11). However, there was an indirect relationship between physical activity habit strength and depressive symptoms, which was mediated via perceived social support (beta=–.13; SE 0.04, 95% CI –0.21 to 0.06). The negative relationship between physical activity habit strength and depressive symptoms was fully mediated by perceived social support.

**Conclusions:**

We suggest that physical activity treatment in people interested in reducing their cardiovascular risk should also embed social support to target depressive symptoms. Internet-based interventions and electronic health may provide a good option for doing so.

**Trial Registration:**

ClinicalTrials.gov NCT01909349; https://clinicaltrials.gov/ct2/show/NCT01909349 (Archived by WebCite at http://www.webcitation.org/73Y9RfdiY)

## Introduction

### Cardiovascular Diseases and Their Burdens

Cardiovascular diseases (CVDs) are the most prominent group of chronic diseases contributing to the global burden of disease. In Germany alone, CVDs, such as heart attacks, coronary heart disease, or strokes, accounted for around 40% of all deaths and 15% of inpatient hospital treatment in the year 2015 [1,2]. CVD is often caused by a person’s lifestyle: besides smoking [3], high blood pressure [4], a diet rich in fat and salt [5,6], and especially physical inactivity [7] are part of the most far-reaching modifiable lifestyle-related risk factors. Meta-analyses of systematic reviews conclude that even 150 min of moderate physical activity per week can reduce CVD mortality by approximately 25% [8,9]. Thus, national and international prevention and intervention programs are often based on these recommendations [10,11].

### Cardiovascular Diseases and Depression

Common concomitant mental illnesses of CVD are depressive symptoms, depression, and anxiety disorders, and depression, in particular, significantly worsens the prognosis of those who are already ill [12]. In a Europe-wide study with over 8500 coronary heart disease patients, it was found that about 25% of those examined had a clinically relevant depression [13]. For example, a person suffering from heart failure may also suffer from a depressed mood as a result of, for example, a prolonged stay in hospital or rehabilitation, surgery, or uncertainty of outcome after a heart attack. In contrast, in a representative sample of 7988 healthy people, aged 18 to 79 years, only 8.1% reported depressive symptoms [14].

Many factors suggest a multicausal relationship between depressiveness and CVDs, which can be interpreted in both directions [15]. Depressiveness and depressive symptoms itself represents a risk factor both for the development of CVD [12] and for the course of patients already suffering from CVD [16]. For example, depressive symptoms in terms of lack of motivation and loss of interest can minimize motivation to implement healthy behaviors [17]. This can, for example, result in moving less and paying less attention to a healthy diet and, thus, might increase the risk of further CVD despite a previously healthy lifestyle. The behavioral treatment approaches in CVD patients, therefore, focus not only on reducing risk behaviors and promoting health behaviors [18] but typically also on depressive symptoms [19,20]. Therefore, the relationship between depressive symptoms and lifestyle factors should be further investigated. This study will focus on one of the main lifestyle factors, namely, physical activity.

### Physical Activity and Its Preventive and Rehabilitative Benefits

Regular physical activity behavior has already been shown to be beneficial in reducing depressive symptoms in clinical depression [21,22], in people with mild depressive disorders [22,23], and in people with long-term stress [24].

Researchers explain the positive effect of physical activity on mental health using, among others, a *bio-psycho-social causal model*. The biological or neurophysiological perspective assumes that the increased release of messenger substances in the brain has a positive effect on a person’s mood [25,26]. The psychological argumentation of Vancampfort et al [27] is based, in particular, on the role of self-efficacy maintenance. According to the authors, physical activity can improve self-efficacy and help overcoming learned helplessness, thereby reducing depressive symptoms. A distraction of anxiety-triggering situations or the interruption of *brooding loops* can also play an important role [28].

Changing physical activity to be more habitual is a desired goal in primary and secondary prevention of CVDs because once a behavior has become habitual, it requires less conscious effort and relapses become less likely [29,30]. Habituation of healthy behavior may be the final phase in the health behavior change chain, whereby the behavior has stabilized and its strength has plateaued [29]. There is also evidence that habit predicts future behavior and health outcomes better than intention [31]. Thus, instead of investigating behavior, this study will focus on habituation.

### Social Support as an Important Factor for Health

Despite evidence regarding the relationship between physical activity and depressive symptoms [32,33], the mediating mechanisms of this relationship are so far rather unexplored. In addition to neurophysiological and psychological changes, regular physical activity seems to be accompanied by changes in perceived social support and the mobilization of support, which are particularly important for people with depressive symptoms.

Perceived social support, in the form of interpersonal relationships and interactions, is considered an important factor for physical and mental health. Social interactions, as experienced in group activities, can help people to cope with stress and overcome challenges better [34]. Fehres and Pauly [35] describe group-based physical activity (eg, in an association) as a “place of open social communication, also with topics that go beyond sport,” such as the individual state of health. A high level of perceived social support, because of regular participation in walking, swimming, or heart training groups, may, therefore, also have an impact on depressive symptoms in individuals. For instance, physical activity in a group can provide positive feedback or help to cope with physical challenges (mastery experience), thus increasing self-esteem [36,37].

One’s own physical activity behavior may also have to be related to the physical activity behavior of one’s own social environment (eg, partner and friends). The use of physical activity and exercise therapy in the prevention and rehabilitation of CVDs can only succeed if the needs of the target group are identified and precisely addressed in appropriate support measures. As physical activity behavior depends, among others, on individual psychosocial determinants such as self-efficacy, the level of depression, or perceived social support, appropriate measurements of these determinants are essential to personalize treatment. Thus, this study aims to investigate the needs of the target group in terms of identifying psychosocial determinants that are relevant in the promotion of health behavior and mental health. In particular, this study aims to unveil the relationship between physical activity habit strength, depressive symptoms, and perceived social support.

### Hypotheses

In this study, we examined the following 2 hypotheses: (1) physical activity habit strength is negatively correlated with depressive symptoms and (2) the negative correlation between physical activity habit strength and depressive symptoms is mediated by perceived social support.

## Methods

### Study Protocol

A detailed description of the study protocol has been described before [38,39]. This study presents secondary analyses of data obtained from the baseline measurement. However, this is the first time depressive symptoms and perceived social support were investigated within this study sample. For this study, only a summary of the study methodology and procedures relevant for this study are provided.

### Study Design, Procedure, and Participants

This is a cross-sectional study using baseline data taken from a randomized controlled trial that was used to investigate the effectiveness of a Web-based intervention to promote physical activity and fruit and vegetable intake [38,39].

The study received ethical approval by the Deutsche Gesellschaft für Psychologie (German Psychological Society; EK-A-SL022013) and the medical ethics committee of Atrium Medical Centre Heerlen in the Netherlands (12-N-124).

The recruitment took place between 2013 and 2015 in cardiac rehabilitation facilities and heart training groups in Germany and the Netherlands. In addition, we called for participation via internet platforms on diabetes and CVDs as well as via an email invitation from 2 research agency’s Web-based panels in Germany and the Netherlands. The inclusion criteria were as follows: (1) age between 20 and 85 years, (2) no contraindications for physical activity and fruit and vegetable consumption, (3) having an interest in improving physical activity and fruit and vegetable consumption, (4) sufficient reading and writing skills in the relevant language (German or Dutch), and (5) computer literacy and internet access. Participation in the study was voluntary, and data were anonymized. Participants were informed about the aims of the study and provided informed consent before participation. In total, 790 participants from Germany (371/790, 47.0%) and the Netherlands participated in this internet-based study.

### Instruments

The internet-based self-report data collection was done via a Web-based questionnaire, which was programmed using the content management system Tailorbuilder. The questionnaires were used in German and Dutch, depending on the country the participants were recruited in.

#### Sociodemographic Variables

We assessed various sociodemographic information such as gender (1=male and 2=female), year of birth, country (1=Netherlands and 2=Germany), employment status (1=working part-time, 2=working full-time, 3=in training, 4=unemployed, 5=retired, and 6=housewife or husband), marital status (1=single, 2=close relationship but not living together, 3=close relationship and living together, 4=marital partnership or common law marriage, 5=divorced, and 6=widowed), and highest level of education (1=no school graduation, 2=primary school education, 3=secondary school education, 4=vocational school graduation, 5=university entrance diploma, and 6=other) in the baseline questionnaire. The participants additionally reported body height and body weight to calculate their body mass index (BMI).

#### Depressive Symptoms

We obtained depressive symptoms via the Center for Epidemiologic Studies Depression Scale-Revised 10-item (CESD-R-10) by Eaton et al [40] (Cronbach alpha=.79) that covers the domains depressed affect, positive affect, somatic complaints, and interpersonal problems. The CESD-R-10 consists of 10 statements, each of which is rated by the participants on a 4-point scale, ranging from 0 (rarely or none of the time) to 3 (most of or all the time). The values of these response categories were reversed for the positive and added to a sum score. The sum scores provided by the participants ranged from 0 to 30. Higher values indicate a higher level of depressive symptoms. To categorize the participants into either depressed or not depressed, we used a cut-off value of 10, which is proposed by Eaton et al [40].

#### Physical Activity Habit Strength

The strength of habit for physical activity (Pearson *r*=.88) was measured with an abbreviated version of the Self-Report Habit Index by Verplanken and Orbell [41,42] and included the following 2 items: “Being physically active for at least 30 minutes on 5 days a week is something that...” (1) “has become a confirmed habit” and (2) “I do without thinking about it.” In all analyses, the mean of these 2 items was used as the dependent variable.

#### Perceived Social Support

Perceived social support [43] was measured with the following 3 items: “My partner helps me to be physically active,” “My family helps me to be physically active,” and “Friends or peers help me to be physically active” (Cronbach alpha=.79). Study participants indicated all items on Likert scales ranging from *1=not true* to *7=exactly true*. In all analyses, the means of these 3 items were used as the independent variable.

### Data Analysis

Data analysis was done with SPSS version 24. The mediation analysis was performed using a SPSS PROCESS version 2 16.3 Macro by Hayes [44,45]. We followed the principles of mediation analysis as described by Baron and Kenny [46]. Our model describes path *c* as the total direct effect of physical activity habit strength regressed on depression. Path *a* is described as the direct effect of physical activity habit regressed on perceived social support and path *b* the direct effect of perceived social support regressed on depression. The total effect *c* is the sum of the direct and indirect effect, that is, *c*=*a* * *b* + *c’*. A reduction of the direct effect *c*, compared with *c’*, indicates a mediation effect of perceived social support. As indirect effects violate the assumption of normal distribution, we used a bootstrapping approach (10,000 bootstrap samples) to provide robust estimates of 95% CIs of the standardized effects. Gender, country of birth, BMI, highest educational status, and age were included as control variables. The level of statistical significance was set at *P*<.05. All reported *P* values are two-tailed. We used no statistical measures to correct for multiple testing.

## Results

### Participation and Sample Characteristics

The mean age of the participants was 50.9 years (SD 12.2; range 20-84), 62.9% were female (497/790), 66.3% (524/790) were married, and 72.0% (569/790) were working full-time or part-time. The mean BMI was 27.5 (SD 5.0; range 17.9-47.3). In total, 38.1% (301/790) of the participants were categorized as depressive. Baseline differences between German and Dutch participants regarding age, gender, and BMI have been reported and critically discussed before [47].

### Intercorrelations Between Study Variables

Intercorrelations between the main study variables were calculated with Bonferroni corrections (*K*=4, *P*<.01). Table 1 reports intercorrelations, ranges, as well as means and SDs. Physical activity habit strength was negatively related to depressive symptoms (*R*=−.13, *P*=.006), and depressive symptoms were negatively interrelated with perceived social support (*R*=−.17, *P*=.004; Table 1).

Age was positively associated with physical activity habit strength (*R*=.16, *P*=.004) and BMI (*R*=.12, *P*=.01). This means that older participants reported stronger physical activity habit strength and a higher BMI. Among our sample, BMI and depressive symptoms were not significantly related (*R*=.08, *P*=.36). There was a small negative association between BMI and physical activity habit strength (*R*=−.10, *P*=.001), indicating that those persons with a higher BMI had lower physical activity habit strength. Finally, physical activity habit strength was positively associated with perceived social support (*R*=.39, *P*<.001), which means that those with stronger habit strength perceived more social support regarding their physical activity. To control for shared variance between the variables and to test for potential psychological mechanisms, a regression analysis was computed next.

### Direct and Indirect Effects of Habit Strength on Depressive Symptoms via Perceived Social Support

**Figure 1:**
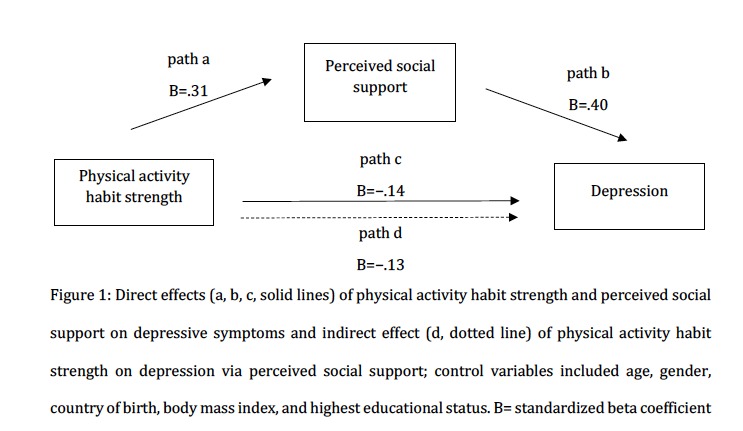
Direct effects (a, b, c, solid lines) of physical activity habit strength and perceived social support on depressive symptoms and indirect effect (d, dotted line) of physical activity habit strength on depression via perceived social support; control variables included age, gender, country of birth, body mass index, and highest educational status. B=standardized beta coefficient.

The regression analysis was employed to test hypothesis 1 and hypothesis 2 in a mediation model. As shown in Figure 1, physical activity habit strength significantly related to perceived social support (path a: B=.31, SE 0.03, *P*<.001) while controlling for depressive symptoms.

Perceived social support significantly correlated with depressive symptoms (path b: B=−.40, SE 0.10, *P*<.001): the higher the perceived social support, the lower the reported depressive symptoms.

In contrast to our bivariate results (Table 1), physical activity habit strength was not correlated with depressive symptoms (path c: B=−.14, SE 0.09, *P=*.11) when controlling for perceived social support. However, in accordance with hypothesis 2, there was an *indirect* effect of physical activity habit strength on depressive symptoms via perceived social support (path d: B=−.13; bootstrapped SE 0.04, 95% bootstrapped CI −0.21 to −0.06). This means that the negative relationship between physical activity habit strength and depressive symptoms was fully mediated by perceived social support. In other words, physical activity habit strength and depression only correlated negatively via perceived social support.

**Table 1:** Descriptive information of the sample.

Variable	Correlation, *R*				Range		Mean (SD)
	1. Age	2. BMI^a^	3. Perceived social support	4. Depressive symptoms (continual)			
1. Age	—	—	—	—		20-84	50.9 (12.2)
2. BMI	.12^b^	—	—	—		17.9-47.3	27.5 (5.0)
3. Perceived social support	.06	<.01	—	—		1-7	3.8 (1.6)
4. Depressive symptoms (continual)	.08	.08	−.17^b^	—		0-25	10.3 (4.0)
5. Physical activity habit strength	.16^b^	−.10^b^	.39^b^	−.13^b^		1-7	3.5 (1.8)

^a^BMI: body mass index.

^b^Correlation (*R*) significant at *P*<.05.

## Discussion

### Objective of the Study

In the prevention and rehabilitation of CVD, the presence of comorbid depressive symptoms remains a factor that should not be neglected, especially when it comes to motivation to adopt or maintain physical activity. In this internet-based study, the relationship between physical activity habit strength and depressive symptoms in 790 people seeking to reduce their cardiovascular risk was investigated, and the explanatory social-cognitive mechanisms of this relationship were analyzed.

### Discussion of Findings Regarding the Hypotheses

Matching our first hypothesis, there was a bivariate correlation between physical activity–related habitual strength and depressive symptoms, showing that the stronger the physical activity habit strength, the lower the depression scores. It is conceivable that physical activity can improve self-esteem and overcome learned helplessness and, thus, alleviate depressive symptoms [28] or serve as a distraction from anxiety-inducing situations [32,33]. Exercise therapy could be a good complement to psychotherapeutic or pharmacological treatment actually provided in a Web-based format.

In line with hypothesis 2, the relationship between physical activity habit strength and depressiveness was mediated by the perceived social support of the study participants. Physical activity often takes place in a social setting, for example, with training partners, exercise instructors, physiotherapists, or other persons in the form of, for example, heart training groups. Physical activity may also have an influence on the level of physical activity of one’s own social environment, for example, one’s partner and friends. Studies revealed that perceived social support from significant others (eg, family and friends) is positively related to physical activity [48]. Differences in health behaviors that are relevant to CVD prevention and rehabilitation can also be because of the impact of the partner or close family member. This has already been shown for smoking behavior and body weight [36], fruit and vegetable consumption, and alcohol consumption [37]. Thus, the lifestyle of one’s partner can significantly influence one’s own lifestyle through model learning (eg, social cognitive theory [49]), setting the subjective norm (cf. theory of planned behavior [50]), and providing or inhibiting social support. The physical activity behavior of a partner can either adapt to one’s own during the partnership or determine the choice of partner in advance. The negative correlations between physical activity habit strength and depressive symptoms can, therefore, probably be explained by mutual perceived support and the shared experience of (physical activity) successes and failures [47].

According to clinical experience, the use of exercise therapy as a structured antidepressive treatment often fails because of a patient’s lack of motivation or their limited ability because of illness [51,52]. It might be worth trying to implement social support from other sources such as peers in internet-based interventions to increase the effects of social support and help to change health behavior among people with smaller social networks. In times of increasing prevalence of single homes, providing such social support or facilitating social support by digital devices appears contemporary.

### Limitations and Implications for Future Research

Our study has several limitations that need to be taken into account and discussed. First, because of the cross-sectional nature of this study, only interrelations, but no causal effects, could be investigated. Future randomized controlled trials with larger sample sizes and additional measurement points can provide information on the direction and sequencing of the effect.

Second, the sample is very heterogeneous in terms of the individual health status of the patients. Although the desire to promote their own heart health was considered a prerequisite for participation, the patients reported very different diseases of their cardiovascular system that could have influenced their depressive symptoms and their physical activity behavior. A medical control variable (such as type and severity of the disease) is recommended for future research.

Third, the measure of physical activity habit strength must be addressed. The self-report habit index we used has good internal consistency [39,41,42]. However, for future studies, because of possible memory effects or social desirability [53,54], objective markers for physical activity such as pedometers [54] or fitness trackers are recommended to be used in addition to the habit measure. A direct indicator of physical activity should be taken into account, and ideally, a distinction should be made between the intensity of the activity (light, moderate, and strenuous): a review by Firth et al [17] showed that strenuous physical activity was more effective in reducing depressive symptoms than moderate or light physical activity. This needs to be taken into consideration for future research.

Fourth, although the correlation between physical activity and perceived social support is supported statistically within this study, this topic needs more attention in future internet research and interventions for patients seeking to decrease their cardiovascular risk. Currently, many questions remain, such as which kind of social support is perceived as most beneficial. Emotional support could help to overcome personal barriers such as low motivation, and informal social support might help to find physical activity patterns, which help to maintain this behavior [55]. Furthermore, the source of the perceived social support might have an influence on habit strengths.

Finally, in our study, just under 38.1% (301/790) of all study participants are classified as depressive, whereas the number of depressive patients among the CVD patients in other studies is typically lower at 23% to 30% [12-16,56,57]. Thus, it does appear that depressive patients are well accommodated by internet approaches as the barriers to disclose their own mental health status are perceived as lower. However, it cannot be ruled out that the questionnaire exaggerates the depressive symptoms, and therefore, the results should be interpreted with caution as the study sample is not representative for other groups of patients.

### Conclusions

Our study adds to the literature because the relationship between regular physical activity and depressiveness could be validated, and the importance of social support in this relationship could be taken into account in this regard. The identified needs of the target group can be seen in terms of not only helping patients to adopt a physically active lifestyle and to habituate to it but also to mobilize social support because only then the beneficial effect for depression results.

The bio-psycho-social aspects of physical activity should be used in the prevention and rehabilitation of CVD. There is still a need for further medical internet research, particularly with regard to the type, duration, and intensity of optimal physical training for depressive symptoms and the embedding of social support in the context of individual clinical constellations. Further research should investigate whether physical activity should be included in the therapeutic repertoire for the treatment of depressive symptoms and in the prevention and rehabilitation of CVD, taking patients’ social environment into account, and how an internet approach should look like best.

## Abbreviations

BMI: body mass index
CESD-R-10: Center for Epidemiologic Studies Depression Scale-Revised 10-item
CVD: cardiovascular disease
